# Animal Models of Enterovirus D68 Infection and Disease

**DOI:** 10.1128/jvi.00833-22

**Published:** 2022-07-19

**Authors:** Meghan S. Vermillion, Justin Dearing, Yun Zhang, Danielle R. Adney, Richard H. Scheuermann, Andrew Pekosz, E. Bart Tarbet

**Affiliations:** a Department of Comparative Medicine, Lovelace Biomedical Research Institute, Albuquerque, New Mexico, USA; b J. Craig Venter Institute, La Jolla, California, USA; c Department of Pathology, University of California San Diego, San Diego, California, USA; d Division of Vaccine Discovery, La Jolla Institute for Immunology, La Jolla, California, USA; e W. Harry Feinstone Department of Molecular Microbiology and Immunology, The Johns Hopkins Bloomberg School of Public Health, Baltimore, Maryland, USA; f Institute for Antiviral Research, Department of Animal, Dairy and Veterinary Sciences, Utah State University, Logan, Utah, USA; National Institute of Allergy and Infectious Diseases

**Keywords:** EV-D68, non-polio enterovirus, respiratory enterovirus, acute flaccid myelitis, AFM

## Abstract

Human enterovirus D68 (EV-D68) is a globally reemerging respiratory pathogen that is associated with the development of acute flaccid myelitis (AFM) in children. Currently, there are no approved vaccines or treatments for EV-D68 infection, and there is a paucity of data related to the virus and host-specific factors that predict disease severity and progression to the neurologic syndrome. EV-D68 infection of various animal models has served as an important platform for characterization and comparison of disease pathogenesis between historic and contemporary isolates. Still, there are significant gaps in our knowledge of EV-D68 pathogenesis that constrain the development and evaluation of targeted vaccines and antiviral therapies. Continued refinement and characterization of animal models that faithfully reproduce key elements of EV-D68 infection and disease is essential for ensuring public health preparedness for future EV-D68 outbreaks.

## INTRODUCTION

Human enterovirus D68 (EV-D68) is a non-polio enterovirus that can cause severe respiratory illness, and infection has been linked with a neurologic syndrome known as acute flaccid myelitis (AFM). Over the past 2 decades, the incidence of reported EV-D68 infections has continued to increase worldwide, and periods of heightened detection have coincided with biennial outbreaks of AFM. Under normal epidemiological conditions, an EV-D68 outbreak was predicted to have occurred in 2020, though significant social distancing measures and mask mandates implemented during the COVID-19 pandemic likely dampened its magnitude (1). In light of the uncertainty around the timing of the next EV-D68 outbreak, the Centers for Disease Control (CDC) cautions health providers to remain vigilant as restrictions associated with the pandemic are lifted (2).

Phylogenetic analyses of contemporary versus prototype EV-D68 strains have revealed significant genetic viral evolution over time. Studies *in vitro* have characterized several functional impacts of these changes on host cell receptor usage and viral RNA metabolism that may contribute to expanded tissue tropism and increased virulence *in vivo* (reviewed in reference 3). Still, there are significant gaps in our understanding of EV-D68 pathogenesis *in vivo*, including knowledge of target cell types, mechanisms of systemic viral dissemination and tissue-specific infection, host innate and adaptive immune responses, and the relative contributions of both virus- and host-specific factors in disease progression and severity. Expanding this knowledge base is critical for development and evaluation of candidate EV-D68 vaccines and therapies. Central to these efforts is the development and characterization of animal models of EV-D68 infection that recapitulate elements of human pathogenesis and disease. Here, we review the current published animal models of EV-D68 infection, highlighting the advantages and disadvantages of each and summarizing their relevant contributions to the field. Further, we discuss some of the limitations of the available models and consider alternative strategies and future directions for animal-based EV-D68 research.

## HISTORY OF RESPIRATORY ENTEROVIRUSES

The *Enterovirus* genus within the *Picornaviridae* family includes seven human viral species: four human enterovirus species (A to D) and three human rhinovirus species (A to C). EV-D68 was first discovered in 1962, but detection was infrequent until 2014, when the Unites States experienced a nationwide outbreak of EV-D68-associated respiratory disease, resulting in 1,153 reported cases (4), and the emergence of new representative strains within the novel B1 subclade (5). Within the viruses isolated from this outbreak, six polymorphisms were identified that were associated with the AFM syndrome, suggesting a function in enhancing neurovirulence potential (6).

**FIG 1:**
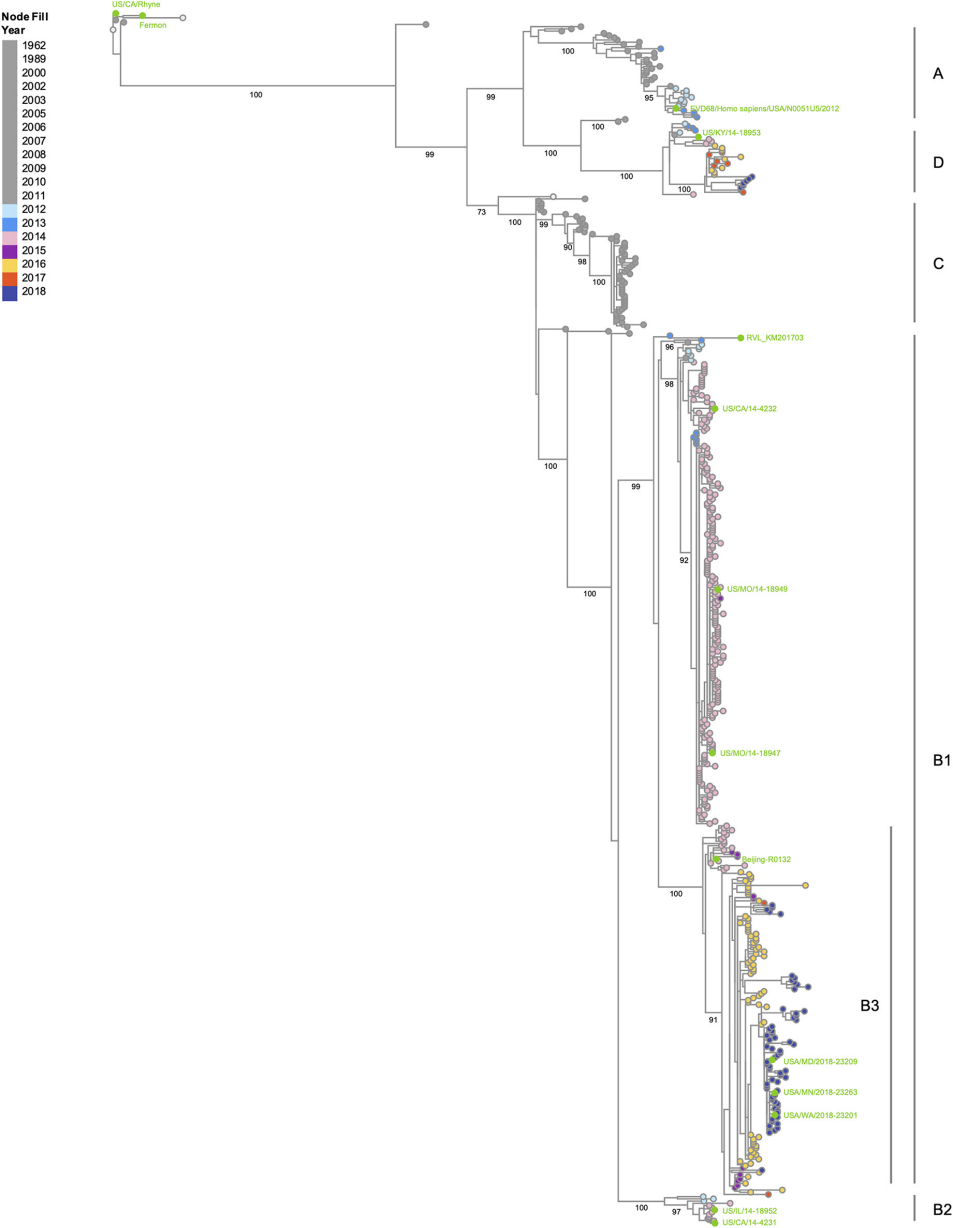
Phylogenetic tree of EV-D68 isolates based on VP1 nucleotide sequences. All available VP1 nucleotide sequences from complete genomes were retrieved from the NIAID Virus Pathogen Database and Analysis Resource (ViPR) site (https://www.viprbrc.org/brc/home.spg?decorator=picorna_entero) (94) on 25 January 2022. EV-D68 isolates used in this review were also included in the data set. The retrieved sequences were aligned using the MUSCLE algorithm on the ViPR site. The resulting alignment was inspected for sequence and alignment quality. A phylogenetic tree was computed using RAxML (bootstrap replicates of 100) and then visualized in Archaeopteryx.js via the ViPR site. Bootstrap support values of 70% or higher are shown for major branches. Tree nodes are color-coded by year of isolation, with isolates included in this review labeled in lime green. Clade classifications are based on bootstrap values of 99% and previous studies (95–97). The tree shows four major clades (A, B, C, and D). Clade B is split into subclades B1 and B2, with B1 containing a subclade B3 referenced in other studies (96).

Since the 2014 EV-D68 outbreak in the United States, the incidence of reported infections has increased, with outbreaks recurring in 2016 and 2018. With each outbreak, EV-D68 has continued to acquire mutations resulting in four major genetic clades—A, B, C, and D—based on phylogenetic analysis of the VP1 gene (Fig. 1), with clades A and B being most prevalent globally (reviewed in reference 3). Between 2014 and 2018, the proportion of EV-D68-positive adult patients increased significantly, and the prevalence of EV-D68 clade D1 infections was higher in adults than in children (7). The contribution of genetic changes in contemporary EV-D68 strains that correlate with these observed changes in disease presentation and differential age-associated susceptibility is an area of active research.

## EV-D68 PATHOGENESIS, CLINICAL DISEASE, AND IMMUNE RESPONSE

### Pathogenesis.

Cell receptor usage across picornaviruses is diverse, and the specific receptor(s) for EV-D68 remain poorly defined. The first hypothesized receptor for EV-D68 was sialic acid (SA), and studies *in vitro* have demonstrated that prototype EV-D68 and isolates from 2010 to 2011 bind both α2,6- and α2,3-linked SA (8, 9). Some EV-D68 strains isolated from 2012 and onward have also demonstrated SA-independent binding *in vitro* (8, 10), suggesting that more contemporary EV-D68 strains may use alternative cell receptors, possibly influencing tissue tropism and disease. Other proposed attachment factors or coreceptors for EV-D68 include sulfated glycosaminoglycans (11) and the neuron-specific intracellular adhesion molecule 5 (ICAM-5) (12). In addition, reported tropism for white blood cells (13) and detection of virus in the serum of patients positive for EV-D68 by nasopharyngeal swabs (9) suggest that pulmonary immune cells may also be permissive to EV-D68 infection and serve as vehicles for systemic viral dissemination, though peripheral EV-D68 detection is uncommon (14).

Detection of EV-D68 from the cerebrospinal fluid (CSF) of AFM patients is very rare. In a study of 11 children with AFM and detectable EV-D68 RNA in respiratory samples, only one demonstrated presence of EV-D68 infection in the CSF (14). In a separate study of 14 adults with AFM, enterovirus RNA was also only detected in one patient, though antibodies to enterovirus peptides were present in 11 patients (15). Although cross-reactivity of antibodies across enteroviruses is poorly understood (16), this could suggest that central nervous system (CNS) exposures to enteroviruses may be more prevalent than PCR detection of virus would suggest.

### Clinical disease.

EV-D68 can infect both the upper and lower respiratory tract in humans, and respiratory disease is characterized by nonspecific symptoms such as cough, congestion, and sore throat, which are occasionally accompanied by fever, vomiting, and diarrhea (4, 17, 18). Severe respiratory disease secondary to EV-D68 infection is most frequently reported in young children and infants, with asthmatic children disproportionately represented (19, 20). Moreover, prevalence of EV-D68 in adults with comorbidities also increased in the most recent 2018 outbreak (7). Because EV-D68 infection is associated with nonspecific symptoms that overlap other more common respiratory infections, it is likely that the overall prevalence is underestimated. It is also probable that there is a large proportion of clinically silent infections, especially in otherwise healthy adults, further contributing to overall underreporting.

Of particular concern to global public health is the temporal association of contemporary EV-D68 outbreaks with AFM (21–24), which is defined by the CDC as the concomitance of acute flaccid limb weakness confirmed with magnetic resonance imaging (MRI) evidence of a spinal cord lesion largely restricted to the gray matter and spanning at least one spinal segment (25). A total of 120 cases of pediatric AFM were reported coincident with the 2014 EV-D68 outbreak in the United States, followed by 153 cases of AFM in 2016 and 238 cases of AFM in 2018. Although a causal link between EV-D68 and AFM symptoms has still not been unequivocally established, human surveillance data and studies in mice (26) suggest that EV-D68 contributes to the increase in pediatric AFM during outbreak years.

During the 2014 outbreak, it was estimated that approximately 10% of the hospitalized patients with EV-D68 infections were at risk of developing AFM (27, 28). The incubation period for clinical AFM symptoms is estimated to be 5 to 7 days following EV-D68 infection. Neurologic injury is typically preceded by a prodromal phase of respiratory symptoms and fever, followed by a rapid onset of flaccid limb weakness ranging from mild paresis to complete paralysis. There has not been a reported correlation, however, between the severity of respiratory disease and the development of AFM (29). Almost all AFM cases require hospitalization, and between 16 and 28% require intubation and mechanical ventilation (2). Although AFM is rarely fatal, the prognosis for full recovery is poor, and many report persistent muscle weakness and atrophy for months to years (30).

### Immune response.

Evasion of host innate immune defenses is central to infection with pathogenic enteroviruses (31). Studies *in vitro* have suggested that interference with type I interferon (IFN) signaling through the Toll-like receptor 3 (32) and IRF-7 (33) are mechanisms by which EV-D68 evades the host innate immune response, but this has yet to be confirmed *in vivo.* In the lung, the early proinflammatory cytokine and chemokine response to EV-D68 infection has been characterized in both mice (34) and cotton rats (35), prompting the recruitment of inflammatory cells; however, the relevance of specific pathways and cell types to EV-D68 clearance is unknown. Furthermore, the corresponding inflammatory markers of EV-D68 infection in the CNS have not been described. Mechanistic studies are needed to determine the relative contribution of key innate immune responses on protective versus immunopathological response to EV-D68 infection in relevant tissues.

The role of antibodies in protection against EV-D68 infection and disease has been studied in more detail, but important questions of the quality, breadth, and durability of antibody-mediated protection remain unanswered. Surveillance studies in humans have shown very high seroprevalence of anti-EV-D68 antibodies among adults (36), and polyclonal antibodies against EV-D68 are detectable in human intravenous immune globulin (IVIG) (37). Young children are protected early in life from passively transferred maternally derived anti-EV-D68 antibodies but likely become vulnerable to infection around 1 year of age when trough population antibody titers are observed (38, 39). Continued circulation of EV-D68 despite high seroprevalence suggests that, similar to antibodies generated against poliovirus (40), anti-EV-D68 antibodies are not likely to induce sterilizing immunity in the primary site of infection (i.e., the respiratory tract) but may be sufficient to prevent progression to AFM disease. This is corroborated by *ex vivo* studies in human and murine polyclonal sera (16) and in human B cells (41), which showed a broad range of antibody phenotypes and extensive cross-reactivity across different non-polio enteroviruses.

Data generated from challenge studies in animal models are conflicting. Passive immunization studies in mouse models have suggested that hyperimmune sera or purified monoclonal antibodies isolated from challenged animals are protective against homologous challenge in naive animals (42, 43). Moreover, data from these studies suggest that anti-EV-D68 antibodies are broadly neutralizing, and may protect against multiple EV-D68 strains (44, 45). Similar studies in cotton rats, however, have shown that immunization with inactivated EV-D68 confers either no protection or enhanced disease following challenge (35). Treatment of mice with human IVIG has been shown to protect against AFM-like disease following EV-D68 challenge (46), but IVIG treatment in human AFM patients has not been associated with a measurable clinical benefit (4). The timing of antibody administration, as well as the quality and quantity of anti-EV-D68 antibodies, likely contributes to some of the discrepancies in these studies.

The protective role of local and systemic cellular immune responses against homologous or heterologous EV-D68 challenge is largely unknown. Though protection from enteroviral infection is thought to rely most heavily on neutralizing antibody response, the role of T cell responses in EV-D68 recognition and clearance may be harnessed for development of broadly protective immunization strategies. Recent reports using the Immune Epitome Database have shed light on specific conserved regions of the enterovirus polypeptide that can be probed to induce cross-reactive CD4^+^ T cell responses (47), but the role of T-cell responses in protection from infection and progression to AFM have not been studied in sufficient detail.

## ANIMAL MODELS OF EV-D68

Central to the clinical advancement of candidate EV-D68 vaccines and therapies is the availability of standardized animal models that faithfully reproduce elements of human infection and disease. Published models to date have evaluated the infection profiles of both historic and contemporary EV-D68 isolates across each of the major EV-D68 viral clades (Fig. 1 and Table 1). Based on the virus isolate, inoculation route, and animal age/species/strain, models of both neurologic (Table 2) and respiratory (Table 3) disease have been characterized. Here, we summarize the current portfolio of available EV-D68 infection models, highlighting the benefits and limitations of each. This comprehensive overview is intended to guide efforts to refine and standardize existing models and develop additional EV-D68 infection models with expanded or complementary utility.

**TABLE 1:** EV-D68 isolates evaluated in animal models

EV-D68 isolate (GenBank/ViPR strain name)	Origin	Yr	Clade	GenBank accession no.	Animal model(s)	Reference(s)
Fermon	USA	1962	Prototype	KU844179	Cotton rat, ferret, Swiss-Webster mouse, ICR mouse	35, 42, 46, 48, 81
US/CA/Rhyne	USA	1962	Prototype	KU844178	Swiss-Webster mouse	48
EVD68/Homo sapiens/USA/N0051U5/2012	USA	2012	A	KT347280	Cotton rat	35
US/KY/14-18953	USA	2014	D	KM851231	Swiss-Webster mouse, ICR mouse	42, 46, 48
US/MO/14-18949	USA	2014	B1	KM851227	AG129 mouse, cotton rat	35, 41, 62–64
US/MO/14-18947	USA	2014	B1	KM851225	Swiss-Webster mouse, ICR mouse, cynomolgus macaque, pigtailed macaque and African green monkey	42, 44, 48, 60, 84
US/CA/14-4232	USA	2014	B1	KU844180	Swiss-Webster mouse	48
US/CA/14-4231	USA	2014	B2	KU844181	Swiss-Webster mouse	48
US/IL/14-18952	USA	2014	B2	KM851230	Swiss-Webster mouse, cynomolgus macaque, pigtailed macaque and African green monkey	46, 48, 84
Beijing-R0132	China	2014	B3	KP240936	KunMing, NIH, C57BL/6, ICR and BALB/c mouse	58
RVL_KM201703	China	2017	B1	MG991260	Rhesus macaque, C57BL/6 mouse	43, 45
USA/MD/2018-23209	USA	2018	B3	MN246002	Rhesus and cynomolgus macaque	84
USA/MN/2018-23263	USA	2018	B3	MN246026	Rhesus and cynomolgus macaque	84
USA/WA/2018-23201	USA	2018	B3	MN245994	Rhesus macaque	84

**TABLE 2:** Animal models of EV-D68 neurologic disease^*a*^

Species	Strain	Immune status	Age (days)	EV-D68 isolate	Clade	Inoculation route	Inoculation titer	Paralytic disease	Frequency (%)	Mortality (%)	Onset of disease (dpi)	Reference
Mouse	Swiss-Webster	Immunocompetent	2	US/KY/14-18953	D	i.c.	2.00E+06	Pa	47	NS	NS	48
				US/IL/14-18952	B2		5.00E+07	Pa	100	NS	3–5	
				US/CA/14-4232	B1		1.00E+05	Pa	33	NS	NS	
				US/CA/14-4231	B2		3.00E+07	NPa	0	NS	NA	
				US/MO/14-18947	B1		5.00E+06	Pa	52	NS	3–9	
				Fermon	Prototype		8.00E+06	NPa	0	NS	NA	
				Rhyne	Prototype		1.00E+07	Rarely Pa	6	NS	NS	
				US/MO/14-18947	B1	i.m.	1.00E+05	Pa	100	NS	2–4	
						i.n.	2.00E+05	Rarely Pa	3	NS	8–10	
						i.p.	1.00E+05	Rarely Pa	5	NS	5	
Mouse	ICR	Immunocompetent	1	Fermon	Prototype	i.p.	2.00E+06	NPa	NA	0	NA	42
				US/KY/14-18953	D			Pa	NS	20	5–10	
				US/MO/14-18947	B1			Pa	100	100	2–7	
			5					Pa	100	100	3–11	
			7					Pa	NS	70	3–9	
			9					Pa	NS	50	4–10	
			12					NPa	NA	0	NA	
			1				1.20E+05	Pa	100	100	3–10	
							7.80E+03	Pa	NS	70	5–12	
							3.00E+01	Pa	NS	30	5–14	
							1.00E+01	Pa	NS	10	7–11	
Mouse	Swiss-Webster	Immunocompetent	2	US/IL/14-18952	B2	i.m.	1.00E+01	Pa	NS	0	4–9	46
							1.00E+02	Pa	NS	5	3–7	
							1.00E+03	Pa	NS	18	2–5	
							1.00E+04	Pa	NS	33	2–3	
Mouse	C57BL/6	Immunocompetent	Neonatal^*c*^	Beijing-R0132	B3	i.p.	1.00E+07	NS	NS	0	NS	58
	ICR							Pa	NS	100	NS	
	KM							–^*b*^	NS	0	NA	
	NIH							–	NS	0	NA	
	BALB/c							Pa	NS	100	2–4	
							5.00E+06	Pa	NS	82	3–5	
							2.50E+06	Pa	NS	65	4–6	
							1.25E+06	Pa	NS	40	4–7	
Mouse	AG129	IFN-α/β/γR^–/–^	10	US/MO/14-18949 (MP30pp)	B1	i.p.	1.58E+05	Pa	50	17	3–6	64
							5.01E+05	Pa	50	17	4–6	
							1.58E+06	Pa	83	66	3–7	
							5.01E+06	Pa	100	100	3–6	
Mouse	AG129	IFN-α/β/γR^–/–^	10	US/MO/14-18949	B1	i.p.	6.31E+06	Pa (ipsilateral)	100	NS	6–8	62
			5	US/MO/14-18949 (MP30)		i.n.	3.16E+06	Pa (bilateral)	50	NS	8 (peak)	

a i.c., intracranial; i.m., intramuscular; i.n., intranasal; i.p., intraperitoneal; dpi, days postinoculation; MP30, mouse-adapted through 30 serial passages in AG129 mice; MP30pp, plaque-purified MP30; NPa, nonparalytogenic; Pa, paralytogenic; NS, not specified; NA, not applicable.

b –, No clinical disease.

c Exact age not specified.

**TABLE 3:** Animal models of EV-D68 respiratory disease^*a*^

Species	Strain	Immune status	Age	EV-D68 strain(s)	Clade	Inoculation route(s)	Inoculation titer	Disease	Viral load assessment	Reference
Mouse	BALB/c	Immunocompetent	8–10 wks	NS 2014 isolate	Unknown	i.n.	5.00E+06	Airway inflammation	RT-PCR: lung	34
		HDM sensitized						Airway inflammation, airway hyperresponsiveness	ND	
Mouse	AG129	IFN-α/β/γR^–/–^	4 wks	MO/14-18949 (MP30pp)	B1	i.n.	3.16E+04	Lung pathology and increased Penh	CCID_50_: lung and blood	64
Mouse	AG129	IFN-α/β/γR^–/–^	4 wks	MO/14-18949	B1	i.n.	3.16E+06	Mild lung pathology	CCID_50_: blood, lung, liver, kidney, spleen	63
				MO/14-18949 (MP10)			3.16E+06	Mild lung pathology and inflammation		
				MO/14-18949 (MP20)			3.16E+06	Mild lung pathology and inflammation		
				MO/14-18949 (MP30)			3.16E+06	Moderate lung pathology and inflammation		
				MO/14-18949 (MP30pp)			3.16E+06	Moderate lung pathology, inflammation, and increased Penh	CCID_50_: blood, liver, kidney, spleen, spinal cord, brain, leg	
							1.00E+06	Increased Penh	CCID_50_: lung, liver, kidney, spleen	
							3.16E+05	Increased Penh		
							1.00E+05	Increased Penh		
							3.16E+04	Increased Penh	CCID_50_: blood, liver, kidney, spleen, spinal cord, brain, leg	
Cotton rat	NA	Immunocompetent	4–6 wks	Fermon	Prototype	i.n.	1.00E+6.0	–^*b*^	TCID_50_: lung and nose	35
	NA	Immunocompetent	4–6 wks	US/MO/14/18949	B1	i.n.	1.00E+6.0	–	TCID_50_: lung and nose	
	NA	Immunocompetent	4–6 wks	VANBT/1	A	i.n.	1.00E+6.0	–	TCID_50_ and RT-PCR: lung and nose	
Ferret	NA	Immunocompetent	NS	Fermon	Prototype	i.n.	1.00E+4.5	Infrequent nasal discharge and cough, and decreased wt gain	RT-PCR, feces, nasal wash, throat swab, blood, lymph node, lung	81
Mouse	C57BL/6	Immunocompetent	Neonatal (exact age NS)	Beijing-R0132	B3	i.p.	1.00E+07	Unspecified mild disease	ND	58
	ICR							Dyspnea		
	KM							–		
	NIH							–		
	BALB/c							Dyspnea	RT-PCR: brain, heart, GI, kidney, liver, spleen, lung, muscle, spinal cord, blood	
							5.00E+06	Dyspnea	ND	
							2.50E+06	Dyspnea		
							1.25E+06	Dyspnea		
Nonhuman primate	Cynomolgus macaque	Immunocompetent	8–12 mo	US/MO/14-18947	B1	i.n./i.t.	4.00E+07	Mild and transient respiratory and gastrointestinal signs; inconsistently reported	RT-PCR and TCID_50_: nasal swabs, BALF, CSF	84
				US/IL/14-18952	B2		4.00E+07			
				USA/MD/2018-23209	B3		4.00E+06			
				USA/MN/2018-23263	B3		4.00E+06			
	Rhesus macaque			USA/MD/2018-23209	B3		4.00E+06			
				USA/MN/2018-23263	B3		4.00E+06			
				USA/WA/2018-23201	B3		4.00E+06			
	Pigtailed macaque			US/MO/14-18947	B1		4.00E+07			
				US/IL/14-18952	B2		4.00E+07			
	African green monkey			US/MO/14-18947	B1		4.00E+07			
				US/IL/14-18952	B2		4.00E+07			

a HDM, house dust mite; i.n., intranasal; i.t., intratracheal; RT-PCR, reverse transcription-PCR assay; CCID_50_, median cell culture infectious dose assay; TCID_50_, median tissue culture infectious dose assay; GI, gastrointestinal tract; BALF, bronchoalveolar lavage fluid; CSF, cerebrospinal fluid.

b –, No clinical disease.

### Mouse models.

The vast majority of *in vivo* EV-D68 infection studies have been performed in mice, in which the selection of strain, immune status, age at infection, EV-D68 isolate, inoculation titer, and route of infection are shown to greatly influence the character and magnitude of the resulting disease phenotype. Importantly, mouse models have been instrumental in differentiating paralytogenic from nonparalytogenic EV-D68 isolates (42, 48). The obvious advantages of mouse models relate to cost and availability, genetic characterization and ease of genetic manipulation, standardized laboratory techniques, and species-specific immunologic reagents that facilitate both mechanistic research and high-throughput screening studies. Productive EV-D68 infection and replication in mice, however, is limited to neonatal animals, immune-deficient strains and/or infection with a mouse-adapted virus. Each of these approaches for modeling EV-D68 infection and disease in mice is described below.

**(i) Neonatal mice.** Neonatal mice are routinely used to model viral encephalitis caused by various neurotropic viruses such as flaviviruses (49, 50), alphaviruses (51, 52), arenaviruses (53), reoviruses (54), and other enteroviruses (55–57). In these models, virus is either congenitally transferred or neonates are inoculated within the first week of birth to produce the desired infection and disease phenotype. Following direct intracerebral injection of AFM-associated EV-D68 isolates (e.g., KY/14-18953, IL/14-18952, and MO/14-18947) into 2-day-old Swiss-Webster mice, between 50 and 100% of animals develop paralysis within 3 to 9 days of inoculation. Intracerebral injection of the prototype Fermon EV-D68 isolate, however, did not produce any paralysis (46) in the same model.

Other routes of infection, including intraperitoneal (i.p.), intramuscular (i.m.), and intranasal (i.n.) inoculations are also capable of producing paralytic phenotypes in neonatal mice, albeit with variable frequencies depending on animal age, mouse strain and EV-D68 isolate and inoculation titer, which are not standardized across studies. In ICR mice, i.p. inoculation with 2 × 10^6^ 50% tissue culture infective dose(s) (TCID_50_) of MO/14-18947 resulted in paralytic disease with 100% incidence in animals up to 5 days old; paralysis was observed in 50 to 70% of animals 7 to 9 days old, and no paralytic disease observed in 12-day-old animals (42). In the same study using 1-day-old mice, decreasing inoculation titers of EV-D68 MO/14-18947 resulted in a decreased frequency and delayed onset of paralysis following i.p. injection, but an infection titer as low as 1.0 TCID_50_ was capable of producing neurologic disease (42). In a separate study, i.p. inoculation of 2-day-old Swiss-Webster mice with 10^5^ TCID_50_ of EV-D68 MO/14-18947 resulted in a <5% incidence of paralysis (48), suggesting that a difference of just 1 day in age may significantly impact the resulting disease in this model.

Induction of paralysis following i.m. inoculation of EV-D68 into neonatal mice has also been described; hind-limb i.m. inoculation of 2-day-old Swiss-Webster mice with 10^5^ TCID_50_ of EV-D68 MO/14-18947 resulted in a 100% incidence of paralysis (48), and hind-limb IM inoculation with 10^4^ TCID_50_ of EV-D68 IL/14-18952 resulted in an approximate 60% incidence of paralysis (46). Paralysis following i.m. viral infection was evident between 2 and 4 days postinoculation (dpi) and presented initially in the inoculated limb followed by progression to the contralateral limb and forelimbs. The lack of standardized methods across each study, however, prevents differentiation of the relative impacts of virus isolate versus inoculation titer on disease incidence.

Studies that describe i.n. EV-D68 inoculation in mice are more limited. In 2-day-old Swiss-Webster mice, i.n. inoculation with 2 × 10^5^ TCID_50_ of EV-D68 MO/14-18947 resulted in an incidence of paralysis of only 3%, which was evident between 8 and 10 dpi (48). Although the low frequency of disease in this model is impractical for efficacy studies, the natural infection route and incubation period more accurately mimic human infection. Also, in 2-day-old C57BL/6 mice, i.n. EV-D68 inoculation (RVL_KM201703 isolate) was shown to productively replicate in the lung and produce characteristic lung and brain lesions, which have been used as efficacy readouts for candidate therapies (43).

Tissue viral load following EV-D68 infection of neonatal mice has served as a secondary endpoint in many studies. Quantitative EV-D68 readouts by PCR and cell-based infectious virus assays have revealed a predilection for nervous, muscle, and respiratory tissue (42–44, 46, 48, 58). Interestingly, multiple groups report presence of virus in blood, likely leading to variable levels of viral replication in other tissues, including the heart, intestine, kidney, liver, and spleen (42, 58). After intracerebral inoculation with the paralytic MO/14-18947 EV-D68 isolate, both EV-D68 RNA and infectious virus were readily detectable in spinal cord tissue, corresponding with positive immunostaining for EV-D68 VP2 in the anterior horn that accompanied histopathological evidence of neuronal cell death (48).

One major limitation of neonatal mouse models is their small size, which precludes repeated sample collections and more comprehensive analysis of viral replication kinetics or immune response within individual animals. In addition, caution should be exercised when extrapolating the pathogenesis of neurotropic viruses from neonatal mice to humans, since there are significant differences in the relative timing of CNS development across species (59). Despite these limitations, neonatal mouse EV-D68 infection models represent tractable models for assessing efficacy of candidate vaccines and passive immunization strategies, which can be evaluated either by immunization of pregnant dams and subsequent challenge of neonates (42, 44, 60) or by direct dosing neonates with polyclonal or monoclonal antibody therapies (43, 46). Increased standardization of methods, animal age/strain, virus isolate, inoculation route, and inoculation titer would facilitate comparison of results across studies.

**(ii) AG129 mice.** IFN signaling is part of the innate immune response and is a first-line defense against viral infections. Successful infection of a host relies on evasion of IFN signaling, which can be host specific. In the case of many human viral pathogens, including EV-D68 (33, 61), mouse-specific IFN responses remain successful at inhibiting infection, and wild-type mice are thus not susceptible to infection or disease. AG129 mice, which lack both type I and type II IFN receptors, are permissive to many human viruses, including EV-D68. Compared to immunocompetent mice, in which induction of paralysis necessitates viral inoculation at postnatal day (PND) 1-2 (42–44, 46, 48), paralysis in AG129 mice was achieved following i.p. inoculation with 6 × 10^6^ TCID_50_ of EV-D68 MO/14-18949 at PND 10 with 100% incidence. Paralysis in this model developed in the hind limbs between 6 and 8 dpi, and virus was present in both muscle and spinal cord tissue, with depletion of motor neurons evident by 6 weeks postinfection (62).

In order to enhance infection and disease in the AG129 mouse model, the clinical EV-D68 MO/14-18949 isolate was serially passaged 30 times through 4-week-old AG129 mice. Intranasal challenge of AG129 mice at PND 5 with 3 × 10^6^ TCID_50_ of the mouse-adapted EV0D68 (ma-EV-D68) isolate resulted in respiratory infection with 100% incidence, and forelimb paralysis with 50% incidence, but no evidence of virus in the spinal cord by immunohistochemistry (62). In 4-week-old AG129 mice, i.n. infection with 3 × 10^6^ TCID_50_ of ma-EV-D68 resulted in moderate lung inflammation and pathology, as well as impaired lung function as evidenced by plethysmography (i.e., enhanced pause) measured 6 to 7 days postinfection (63, 64). Although clinical disease in this model was overall mild (i.e., no significant weight loss), and there was no observed mortality, there was marked induction of proinflammatory cytokines in the lung, and EV-D68 titers in the lung, blood, liver, kidney, spleen, muscle, spinal cord, and brain tissue, which can serve as biomarkers of infection and disease to be used as endpoints for efficacy evaluations (63). Using this model of respiratory disease and the PND 10 AG129 model of paralytic disease, a candidate human monoclonal antibody was shown to neutralize EV-D68 and prevent infection and disease (41).

The obvious limitation of using AG129 mice for studying EV-D68 infection is the absence of IFN signaling that biases the model toward enhanced disease. Thus, this model is not ideal for mechanistic studies of innate immune response to EV-D68 or for assessing the efficacy of immunomodulatory treatments. An important advantage over the neonatal mouse models, however, is the ability to infect older (i.e., 10-day- to 4-week-old) animals, which more closely model human children and adolescents (59). Moreover, larger animal size also permits more sophisticated and translational disease endpoints, such as pulmonary function and electrophysiology.

**(iii) Mice with comorbidities.** In an epidemiologic study of the 2014 EV-D68 outbreak in the United States, over half of hospitalized patients confirmed positive for EV-D68 had a history of asthma or reactive airway disease. These patients were also more likely to require intensive care admission and ventilator support (65, 66), suggesting that preexisting respiratory disease is an important risk factor for infection with EV-D68. Understanding the balance between protective versus pathological immune response to EV-D68 infection can inform therapeutic strategies that target exaggerated inflammatory responses that may contribute to more severe disease.

In a BALB/c mouse model of i.n. EV-D68 infection, simultaneous inhaled exposure to house dust mite—to induce an allergic airway response—resulted in increased recruitment of neutrophils and eosinophils to the airways and increased expression of proinflammatory and asthma-associated cytokines, interleukin-17A (IL-17A), CCL11, IL-5, and Muc5AC (34). Moreover, compared to infection with human rhinovirus, EV-D68 infection resulted in greater IL-17A-dependent airway inflammation and hyperresponsiveness that was responsive to anti-IL-17 antibody treatment (34), suggesting anti-IL-17 antibody as a potential therapy for severe EV-D68 respiratory disease in asthmatic patients. Additional studies in animal models of asthma and allergy are warranted to further mechanistic understanding of the inflammatory response to EV-D68 in this population and to determine the need for individualized medicine in patients with comorbidities.

### Cotton rats.

Owing in part to the abundance of α2,6-linked SA receptors in their upper and lower respiratory tract (67), the cotton rat is susceptible to human respiratory viruses, including respiratory syncytial virus, metapneumovirus, rhinovirus, and influenza viruses, where permissiveness to infection can surpass that of mice by >100-fold (68). An additional advantage of the cotton rat is the availability of species-specific reagents for the study of cellular immune response to infection, which has aided the study of vaccine-associated enhancement of respiratory disease (69, 70), as well as immune senescence in aged animals (71, 72).

In the context of EV-D68, Patel et al. demonstrated that the cotton rat was susceptible to infection with both historic and contemporary isolates, albeit with variable tissue titers and inflammatory response profiles. Intranasal inoculation of adult cotton rats with EV-D68 Fermon, EV-D68 VANBT/1, or EV-D68 MO/14/19 revealed that EV-D68 VANBT/1 resulted in the greatest viral titers in the nose and lung. Regardless of the EV-D68 isolate, however, viral replication kinetics were rapid, with peak viral titers detected 10 h postinoculation (hpi) and viral clearance by 48 hpi (35). Likely owing to the limited detection of EV-D68 Fermon and MO/14/19 replication in respiratory tract tissues, seroconversion rate among animals challenged with MO/14/19 and Fermon was 20% and 0%, respectively. Both i.n. and i.m. inoculation of cotton rats with live EV-D68 VANBT/1, however, induced a robust serum neutralizing antibody response, but only i.m. immunization protected against homologous rechallenge (35).

In the cotton rat, i.n. inoculation with 10^6^ TCID_50_ of EV-D68 VANBT/1 resulted in a measurable, but transient chemokine and cytokine response. Based on the panel of proinflammatory genes evaluated (GRO, IFN-β, MCP-1, RANTES, IP-10, Mx-1, Mx-2, IFN-γ, and IL-6), mRNA induction in the lungs peaked between 4 and 24 hpi, with expression of most genes returning to baseline by 48 to 96 hpi. Corresponding lung pathology was evident at both 48 and 96 hpi, but clinical disease was not reported. Interestingly, i.m. immunization with EV-D68 VANBT/1 was shown to exacerbate cytokine induction and lung pathology after homologous challenge despite providing evidence of sterilizing immunity (35), which raises important questions about the potential for antibody dependent disease enhancement that have not been described in murine EV-D68 challenge models.

Cotton rats, in summary, may represent a valuable model of certain aspects of EV-D68 infection and immune response. Pulmonary cytokine induction that is modulated by preexisting immunity provides a unique platform for studying mucosal immune response to candidate EV-D68 vaccines. The rapid viral replication kinetics, however, may preclude assessments appropriate postexposure therapeutic windows for candidate therapies. Moreover, the restricted susceptibility to certain EV-D68 isolates and lack of paralytic disease may limit application of the model to humans. Assessment of the effect of age on disease progression has also not yet been reported in cotton rats, warranting additional studies to potentially expand the utility of this model.

### Ferrets.

Ferrets are widely accepted models of influenza virus infection and pathogenesis, and adult animals are susceptible to human influenza viruses without any prior host adaptation of the virus (73). Ferrets are also susceptible to other human respiratory viruses, including respiratory syncytial virus (74, 75), metapneumovirus (76), and severe acute respiratory syndrome (SARS) coronaviruses (77, 78). Importantly, ferrets are also the gold standard model of aerosol transmission for pandemic viruses, including influenza (79) and SARS coronaviruses (80).

There is only one published study (81) that describes EV-D68 infection in ferrets, which reports replication and disease pathogenesis following i.n. inoculation with the prototype EV-D68 Fermon strain. Overt signs of respiratory illness were absent in the majority of animals, and there was no significant change in body temperature following viral infection. The normal body weight gain experienced by uninfected ferrets, however, was attenuated by approximately 11% in animals infected with EV-D68, suggesting mild clinical disease not associated with other measurable signs of morbidity. Ferrets in this study demonstrated no evidence of paralysis or other neurologic deficits.

Despite mild clinical disease associated with EV-D68 infection in ferrets, both the upper and lower respiratory tract, as well as the gastrointestinal tract, supported robust viral replication, with peak viral load in feces and nasal washes apparent at 5 and 9 days postinfection, respectively. High viral load in lung tissue was also consistently detected from 3 to 9 days postinfection. Viral clearance in the upper respiratory tract was achieved by 14 days postinfection. Viremia was overall low, and detection of viral RNA in peripheral blood was limited to 3 to 5 days postinfection (81).

Histopathological changes associated with EV-D68 infection in ferrets was observed in lung tissue collected 3 and 7 days postinfection, and was characterized by inflammation and diffuse alveolar hemorrhage in the lower respiratory tract, but not in the trachea. Microscopic lesions colocalized with positive immunostaining for the EV-D68 VP1 antigen, as well as with α2,6-linked SA residues in the lung (81), which represent proposed receptors for the EV-D68 Fermon strain and several 2010 to 2011 EV-D68 isolates (8, 9). Whether the ferret is a susceptible host to more contemporary EV-D68 isolates, which have demonstrated capacity for sialic acid-independent infection *in vitro*, remains unknown.

### Nonhuman primates.

Nonhuman primates (NHPs) are the most phylogenetically similar species to humans, and they are the gold standard for preclinical pharmacology and toxicology studies. The relatively large size of NHPs permits higher volume and more frequent sample collections, as well as serial collection of certain specialized samples, such as bronchoalveolar lavage fluid (BALF) and cerebrospinal fluid (CSF), that are not feasible to collect from rodents as a survival procedure. In addition, standard protocols exist for advanced imaging (e.g., MRI) and electromyography in NHPs that can be used to localize spinal cord lesions and characterize the patterns of denervation associated with the AFM phenotype, providing greater translational relevance to human AFM patients. Using these diagnostic tools, studies in NHPs can greatly expand our knowledge of basic EV-D68 pathogenesis, which provides a platform to validate therapeutic endpoints that can translate directly to human clinical trials.

NHPs have been shown to harbor many different simian enteroviruses, with up to 72% amino acid identity to related human enteroviruses (82). Rhesus, bonnet, and cynomolgus macaques have all demonstrated susceptibility to poliovirus infection and associated poliomyelitis following oral viral inoculation (83). Published reports of EV-D68 infection in NHPs are limited to two studies in 6-month-old rhesus macaques, which were reported to be permissive to i.n. infection with EV-D68 (KM Isolate, RVL_KM201703), a 2017 isolate mapped to the B1 clade (Fig. 1). After inoculation, virus was detectable in serially collected nasal swabs, blood, and fecal specimens out to 14 days postinfection, as measured by PCR. Neutralizing antibody response was primarily directed against the EV-D68 VP1, and monoclonal antibodies isolated from EV-D68-infected macaques conferred protection against i.n. EV-D68 challenge in neonatal mice (43). In a similar study, 6-month-old rhesus macaques immunized with a formaldehyde-inactivated EV-D68 vaccine were used for isolation of memory B cells to evaluate *ex vivo* binding and neutralization activity of specific monoclonal antibodies (45). Neither study reported any respiratory or neurologic disease following infection.

The susceptibility of other NHP species to EV-D68 infection and evaluation of infection with AFM-associated EV-D68 strains are areas of active research. Recent studies in our laboratory have compared infection of juvenile (i.e., 8 to 12 months of age) cynomolgus macaques, pigtailed macaques, rhesus macaques and African green monkeys with five different 2014 and 2018 EV-D68 isolates. Infection with 4 × 10^6^ TCID_50_ of EV-D68 (2018 isolates) by a combined i.n. and intratracheal (i.t.) inoculation resulted in transient viral shedding in nasal swabs and bronchoalveolar lavage fluid that was limited to 3 days postinfection. No virus was detected in cerebrospinal fluid. Similar infection with 4 × 10^7^ TCID_50_ of EV-D68 (2014 isolates) resulted in no evidence of viral shedding. There was no indication of differential susceptibility to infection between NHP species, as could be evaluated by the study. Overall seroconversion rate was 25% for the 2014 isolates and 58% for the 2018 isolates. Associated clinical disease was characterized by mild and inconsistent respiratory and gastrointestinal symptoms, and there were no reports of paralysis or other neurologic deficits over the course of the 26- to 28-day observation period (84).

The apparent resistance of NHPs to EV-D68 infection has several possible causes. Of note, SA receptors are not as widely expressed in the respiratory tract of NHPs compared to humans (85), which may impact susceptibility to EV-D68 strains that depend on this for infection. In addition, there is recognized divergence in the magnitude and specificity of virus-induced innate immune response across humans and NHP species, which is a proposed mechanism for differential susceptibility to certain viruses (86). It is also possible that the studies performed to date are insufficient to ascertain NHP susceptibility to EV-D68 relative to humans and other animal models. Based on these studies, however, it is likely that induction of neurologic disease following EV-D68 infection in NHPs will require either direct CNS inoculation and/or blockade of IFN response, similar to the paralytic EV-D68 infection models in mice.

## DISCUSSION AND FUTURE PERSPECTIVES

Currently, there are no approved antiviral drugs or vaccines available for the treatment or prevention of EV-D68 infection and disease. Current recommended therapies for hospitalized AFM patients are anecdotal and consist of supportive care and administrative of IVIG, high-dose intravenous steroids, and antivirals in some cases (87). Treatment is not standardized, since no significant clinical improvement has been demonstrated with any defined therapeutic strategy. Recent studies have identified several candidate antiviral compounds that have demonstrated efficacy *in vitro* (88–90), but confirmation of efficacy in relevant *in vivo* EV-D68 challenge models is lacking. A variety of different vaccine candidates and passive immunization strategies have been developed based on monoclonal antibodies identified from *in vitro* neutralization assays, and several of these candidates have shown promise by mitigating EV-D68 infection and disease in mice. Without a clear understanding of the pathogenesis of EV-D68 infection of the respiratory tract leading to the AFM syndrome, however, it is difficult to predict the translational potential of such strategies in humans.

While *in vitro* studies have significantly expanded our understanding of the mechanisms of EV-D68 cell entry and replication, *in vivo* studies aimed at the pathogenesis and mechanisms of disease induction have yielded variable results (46). Indeed, much of the challenge with modeling the AFM phenotype secondary to a respiratory EV-D68 infection stems from the low incidence of paralytic disease in humans. Likewise, even in the perfect model system, incidence of AFM is predicted to be low, which then obligates enrollment of very large cohorts of animals in order to detect the phenotype in a sufficient number to evaluate outcomes. Artificially increasing disease incidence through various manipulations of the host and/or virus present opportunities to circumvent these barriers to infection. Such strategies have been employed in mouse models of EV-D68 infection, where the use of either neonatal animals, an immunodeficient strain, and/or infection with a mouse-adapted virus results in productive viral replication disease. Acknowledging the limitations of each of these models, they still provide relatively high-throughput and tractable systems for screening candidate EV-D68 treatments. A major challenge in the review of published murine EV-D68 infection models is the lack of standardized methods and reporting, which is problematic for comparing results. Continued refinement and harmonization of these models is important for accurate and efficient identification and screening of candidate EV-D68 treatments.

The evaluation of EV-D68 infection and disease in species other than mice is limited to two published studies in cotton rats and ferrets. Syrian golden hamsters have been used as models of other enterovirus infections, such as coxsackievirus A16 (91) and enterovirus A71 (92), but there are no published models of EV-D68 infection of hamsters. Similarly, though guinea pigs have been used extensively for modeling human respiratory virus infections (93), they have not yet been formally evaluated as a suitable model of EV-D68. Comparative studies to determine species-specific susceptibility of various small and large animal species to both historic and contemporary EV-D68 isolates may help define host factors that influence disease pathogenesis and severity.

Based the current body of EV-D68 literature, endpoints such as clinical paralytic scoring and mortality have predominated as the standards for evaluating efficacy of candidate therapies. Secondary endpoints include tissue viral load in the respiratory tract, muscle and/or CNS. Limited detection of virus in these compartments in both humans and animal models may suggest that direct virus-mediated effects are not primary determinants of clinical disease presentation and severity, but this remains poorly understood. Perhaps the least understood component of EV-D68 pathogenesis and disease is the resulting inflammatory response. Though it is well documented that many patients experience muscle weakness and atrophy that persists for months to years following recovery from EV-D68 infection (30), the mechanisms of these chronic symptoms are not understood. Current paralytogenic EV-D68 mouse models capture only the acute infection and AFM, and owing to the overall high mortality, chronic disease symptoms have not been characterized. It is possible that successful therapeutic strategies for chronic disease may require targeting of both virus- and host-specific factors that drive this pathogenesis. Development of animal models that mimic the chronic disease sequelae is important for determining predictive factors and informing treatment strategies for these scenarios.
